# Mapping the evidence on dementia care pathways – A scoping review

**DOI:** 10.1186/s12877-024-05250-4

**Published:** 2024-08-17

**Authors:** Marianne Saragosa, Evan MacEachern, Mary Chiu, Sean Weylie, Krista Schneider, Elaine R Maloney, Jordanne Holland, Kerry Kuluski, Ani Orchanian-Cheff, Michelle LA Nelson

**Affiliations:** 1 Science of Care Institute, Lunenfeld-Tanenbaum Research Institute, Sinai Health, Toronto, ON M4M 2B5 Canada; 2 Ontario Shores Centre for Mental Health Science, Whitby, ON L1N 5S9 Canada; 3 Faculty of Health Science, Ontario Tech University, Oshawa, ON L1H 7K4 Canada; 4 Alzheimer Society Peel, Mississauga, ON L5G 3N6 Canada; 5 Alzheimer Society of Chatham-Kent, Chatham, ON N7L 5M8 Canada; 6 Family Caregiver, Ontario, Canada; 7 Centre for Addiction and Mental Health, Toronto, ON M6J 1H4 Canada; 8 Baycrest Health Sciences, Toronto, ON M6A 2E1 Canada; 9 Trillium Health Partners, Mississauga, ON L5B 1B8 Canada; 10 Library and Information Services, University Health Network, Toronto, ON M5G 2C4 Canada; 11 Institute of Health Policy, Management and Evaluation, University of Toronto, Toronto, ON M5T 3M6 Canada

**Keywords:** Dementia, Care pathway, Person-centred, Care partner, Scoping review, Patient engagement

## Abstract

**Background:**

One way of standardizing practice and improving patient safety is by introducing clinical care pathways; however, such pathways are typically geared towards assisting clinicians and healthcare organizations with evidence-based practice. Many dementia care pathways exist with no agreed-upon version of a care pathway and with little data on experiences about their use or outcomes. The objectives of the review were: (1) to identify the dementia care pathway’s purpose, methods used to deploy the pathway, and expected user types; (2) to identify the care pathway’s core components, expected outcomes, and implications for persons with dementia and their care partners; and (3) determine the extent of involvement by persons with dementia and/or their care partners in developing, implementing, and evaluating the care pathways.

**Methods:**

We systematically searched six literature databases for published literature in the English language in September 2023 utilizing Arskey and O’Malley’s scoping review framework.

**Results:**

The findings from the dementia care pathways (*n* = 13) demonstrated assistance in dementia diagnostic and management practices for clinicians and offered structured care processes in clinical settings. For this reason, these pathways emphasized assessment and interventional post-diagnostic support, with less emphasis on community-based integrated dementia care.

**Conclusion:**

Future dementia care pathway development can seek the involvement of persons with dementia and care partners in designing, implementing and evaluating such pathways, ensuring that outcome measures properly reflect the impact on persons with lived dementia experience and their care partners.

**Supplementary Information:**

The online version contains supplementary material available at 10.1186/s12877-024-05250-4.

## Background

Presently, more than 55 million people are living with dementia worldwide, with an incidence of 10 million new cases annually [1]. With no cure for dementia, those living with the condition and their care partners require and rely on pharmacological and non-pharmacological interventions, support and resources [2]. Advocates of post-diagnostic support have called on service delivery models that are “one-stop-shop” in nature, consisting of education, case management, legal services, allied health, and culturally appropriate health care [3]. People with dementia and their care partners also seek opportunities to engage in meaningful research [4] and look to clear communication with primary care physicians [5]. As such, an accurate diagnosis facilitates an entry point to treatment and intervention and for families planning and preparing for the future [6]. However, barriers to dementia-related education and support services exist, and they include low knowledge of services, lack of resources (e.g., transportation, financial), values and beliefs, and stigma [7, 8]. Existing research describes system navigation of the formal care systems by care partners as burdensome given the lack of clear and transparent information and resources, fragmentation and an absence of coordination, and unresponsive services to family needs and circumstances [9]. The burden of navigational work manifests in excessive time and energy needed to engage in system navigation and the emotional toll (e.g., confusion, frustration, feeling overwhelmed) placed on individuals and family care partners [10]. For dementia care partners, the disease progression triggers specific care partner support needs corresponding to phases of the caregiving trajectory [11].

One way of standardizing practice and improving patient safety is by introducing clinical care pathways [12]. Clinical pathways are structured, multidisciplinary care plans meant to support the implementation of protocols and clinical management of a defined patient population [13] for a well-defined period [14]. Research suggests that clinical care pathways, when implemented in hospital settings, may be associated with improved quality of care, decreased hospital costs, and increased staff satisfaction [15, 16]; however, such pathways are typically geared towards assisting clinicians and healthcare organizations with evidence-based practice [17, 18]. On the other hand, care pathways are defined as longer and include more facets of the care process, such as discharge from the hospital and after-care [14]. According to Samsi and Manthorpe [19], care pathways or other suggested terms (e.g., critical care pathway, integrated care pathway, etc.) systematically plan, modify or vary patient care and organize follow-up care. Given the promise of clarity, care pathways can offer, and that living with dementia often leads to anxiety and confusion [20], a dementia care pathway can provide reassurance and a clearer image of the prognosis and timeline [19].

Many dementia care pathways exist with no agreed-upon version of a care pathway and with little data on experiences about their use or outcomes [19]. For example, existing dementia care pathways vary in format (i.e., online platform) [21], intended discipline (i.e., nursing) [22], and target population (i.e., prisoners) [23]. Awareness of and efforts for patient and public engagement in improving health service delivery is considered foundational to quality care pathways [24]. Considerable literature indicates patient involvement can lead to positive clinical and patient-level outcomes (i.e., empowerment), quality of care, and the organizational setting (i.e., culture shift) [25, 26]. However, the involvement of persons with dementia and their care partners in developing, implementing, or evaluating such pathways remains elusive. The shift to patient engagement extends to dementia care service design and research [27, 28] and increasingly person-centred care, emphasizing the person behind the patient [29].

Given the growing number of persons with dementia and the potential for dementia care pathways delivering person-centred care, we conducted a scoping review to map and synthesize the dementia care pathway literature. This consisted of identifying and reporting the empirical evidence according to the following objectives: (1) to identify the dementia care pathway’s purpose, methods used to deploy the pathway, and expected user types; (2) identify the care pathway’s core components, expected outcomes, and implications for persons with dementia and their care partners; and (3) determine the extent of involvement by persons with dementia and/or their care partners in developing, implementing, and evaluating the care pathways.

## Methods

We followed the Arskey and O’Malley (2005) six-step approach, including a consultation stage [30]. We reported in adherence with the Preferred Reporting Items for Systematic Reviews and Meta-Analyses extension for Scoping Reviews (PRISMA-ScR) Checklist [31] (Supplemental Sfile 1).

### Step one: developing the research question

To address the objectives of our scoping review, we sought to answer the following question: What are the existing dementia care pathways, their purpose, and core components?

### Step 2: identifying relevant studies

We searched six literature databases for this review in September 2023: Ovid MEDLINE, Ovid Embase, Cochrane Database of Systematic Reviews, Cochrane Central Register of Controlled Trials, PsycINFO, and CINAHL. Our comprehensive search strategy was developed with the assistance of an Information Specialist using a combination of database-specific subject headings and text words for the main concepts of dementia and care pathways. To increase consistency with previously published work in this domain, we adopted a dementia search string from the Cochrane Collaboration [32], included in Supplemental file 2. We also manually reviewed the reference list of relevant papers to identify new articles appropriate to our research question. Final search results were exported to Covidence Reference software (Veritas Health Innovation, Melbourne, Australia) to remove duplicates [33].

### Eligibility criteria

We included studies that discussed or investigated dementia care pathways (or related terminology such as clinical pathway or critical care pathway) in the context of their development, evaluation, or implementation. For this scoping review, the dementia care pathway was broadly defined as an approach to systematically planning, modifying, or varying patient care or organizing follow-up care across four common points: early symptom identification and first service encounters, assessment process, diagnostic disclosure, and post-diagnostic support and appropriate intervention [19]. Our exclusion criteria included care pathways, which focussed on a singular time point (e.g., end-of-life), articles focused on non-dementia populations, commentaries, conference abstracts, or posters that did not provide sufficient information about the care pathway. We limited our search results to articles published using human subjects and in English (see Table 1).

**Table:** 

Included	Excluded
*Population: People living with dementia*	
• Sources that include a participant group who are affected with dementia OR • Sources that focus on the dementia patient population	• Sources in which it is not made explicit that the participant group is affected with dementia OR • Sources that focus on non-dementia patient population
*Concept: Dementia care pathway*	
• Defined as an approach to systematically planning, modifying, or varying patient care or organizing follow-up care across four common points: early symptom identification and first service encounters, assessment process, diagnostic disclosure, and post-diagnostic support and appropriate intervention	• Sources that focus on pathways for end-of-life, responsive behaviours or on one of the four common points *only*
• Sources that are academic literature (i.e., studies, conceptual papers)	• Sources with insufficient details to meaningfully meet the scoping review objectives
• Sources from any geographic region	• Sources without English language full text

### Search terms

See Supplemental file 2.

### Step 3: study selection

The reviewers (MS, EM) screened 100 duplicate titles and abstracts for two rounds to ensure consistency between reviewers. This exercise yielded an inter-rater coefficient of 67% and 87%, indicating a high consistency rate [34]. Reviewers then independently evaluated title and abstract screening and independently completed a full-text review of all eligible articles from the title and abstract screening process. The first author identified grey literature by manually searching Google Scholar for technical reports of dementia care pathways that met eligibility criteria. Our article selection process required no third-party discussions.

### Step 4: charting the data

#### Data extracted

Two independent reviewers (MS, EM) extracted data from the included articles using a data extraction template in Covidence software developed by the research team. The data extraction form included information on the study characteristics (I.e., title, author(s), year and country of publication, journal, and funding sources), study objective/purpose, study design, care pathway components, user type, study outcomes, and implications for persons with dementia and their care partners. We also captured the reported involvement of those affected by dementia in developing, implementing, or evaluating the care pathway.

**Fig. 1:**
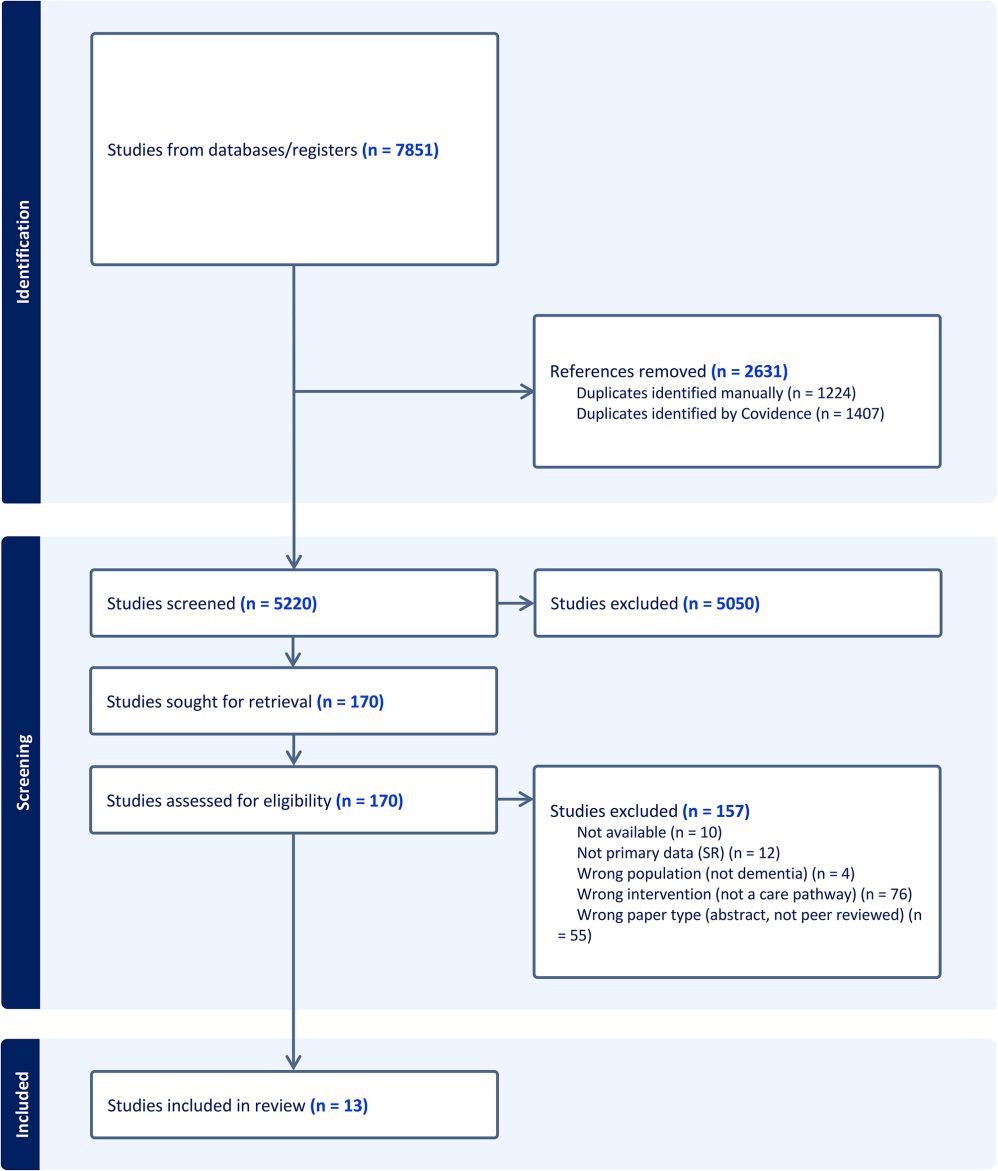
PRISMA (Flow diagram of study selection)

### Step 5: collating, summarizing, and reporting the results

The extraction data was analyzed deductively according to the content analysis method [35] and bound within our research objectives. Using tables and figures, we summarized article characteristics, care pathway purpose, and method used to deploy the pathways and expected users. We synthesized care pathway components, expected outcomes, and implications for the users identified and grouped findings by identified themes. The authors also synthesized the methodological limitations of the included studies. Finally, we reported any reference to user involvement in pathway development, implementation or evaluation processes.

## Results

### Publication characteristics

In total, 7851 articles were identified from our database search; 2631 duplicates were removed. Of the remaining 5220 articles, 170 met the criteria for full-text review. After a full-text review, 13 articles were included, with one additional study from hand-searching (see Fig. 1) [36]. Eight studies reported sample sizes representing 2,568 participants, including program referrals [21, 37–43]. Studies included various populations of interest, including exclusively persons with dementia and family care partners (*n* = 4) [37–40] or physicians, nurses, clinical staff, and aged-care service providers (*n* = 5) [21, 23, 43–45]. Two studies used a triadic approach involving clinicians, persons with dementia, and care partners [39, 42] (see Table 1).

Four studies were geographically located in the United Kingdom [23, 38, 41, 44] and Australia [21, 39, 40, 45], followed by single articles from China [46], Thailand [42], and Switzerland [43]. The study designs reported consist of a mixed methods process evaluation [43], a quantitative pilot study [21], a co-creation and participatory action research [40], a mixed methods study [23], and a technical collaborative action research [42]. A study design was not reported in the remaining nine studies [18, 37–39, 41, 44–47].

**Table 1:** Publication characteristics

Study	Year	Country	Study Design	Population	Sample Size	Average Age	Summary of findings
Aguirre et al.	2022	USA	Program conceptualization and evaluation	Persons with dementia and care partners	1492 (747 patients, 745 care partners)	Patients = 73.3 *±* 9.3 Care partners 61.2 *±* 13.8	Collaborative and interprofessional approaches hold promise for addressing current limitations in dementia care.
Aldridge et al.	2019	United Kingdom	Not reported	Admiral nurses (dementia specialized nurses)	n/a	n/a	The inclusion of Admiral Nursing within primary care networks and integrated care systems models could offer an opportunity to incorporate the specialist clinical skills and knowledge required to affect change and offer services closer to home.
Carter et al.	2021	China	Not reported	Persons with dementia and care partners	n/a	n/a	Cultural awareness, local knowledge and community engagement and education are crucial to the development and implementation of innovative solutions to support dementia care.
Davies & Larner	2010	United Kingdom	Not reported	Persons with dementia and care partners	51 patients (9 from family support workers, 42 from neurologist case records)	60 years (range: 42–78)	Devising an integrated care pathway which adequately addresses disease heterogeneity and patient needs in a slowly progressive disorder is difficult, but ultimately worthwhile to ensure timely diagnosis and access to appropriate care needs
Fitzgerald et al.	2018	Australia	Program conceptualization and evaluation	Persons with dementia and care partners	25 (7 patients, 18 care partners)	n/a	This research project used the “voice of consumers” to develop visualisations of eighteen personal journeys of people living with dementia and their carers. Participants stated that the consumer-centric, visual approach resonated very strongly with them, far more than the written output that they had been presented with in the past.
Forsyth et al.	2020	United Kingdom	Mixed methods study	Prison and community-based services staff	n/a	n/a	Authors developed a care pathway and training materials to provide a framework that prison officers, prisoners, health and social care staff, and other statutory and third-sector organisations can adapt to fit local circumstances.
Goeman et al.	2016	Australia	Program conceptualization	Persons with dementia and care partners	62 (11 care partners)	69 *±* 14	The Specialist Dementia Nurses model of care and Culturally and Linguistically Diverse dementia care pathway addresses current healthcare system service gaps by providing culturally and linguistically diverse communities with health and social care services that are culturally appropriate.
Hampel et al.	2022	USA	Not reported	Persons with dementia and care partners	n/a	n/a	The conceptualization of Alzheimer’s Disease as a clinical–biological construct and the emerging biomarker-guided pathway-based treatments targeting Alzheimer’s Disease-associated pathophysiology highlight the importance and urgency of developing and implementing a global framework for the next-generation Alzheimer’s Disease clinical care pathway.
Hean et al.	2010	United Kingdom	Program conceptualization and evaluation	Case referrals	478 (cases diagnosed with dementia)	n/a	The Mid-Essex Memory and Support Service approach appears to meet its stated aims and has improved the service for people with dementia, their carers and families through its streamlined and integrated pathway.
Lhimsoonthonet al.	2019	Thailand	Not reported	Dementia care pathway stakeholders	346 (14 primary care providers, 21 community health volunteers, 319 older people, and 12 care partners of people with dementia)	n/a	In preparing to face a growing aging population with dementia, nurses and primary care providers should take leading roles in developing dementia care services by implementing this Dementia Care Service pathway at the primary care settings in the future.
Morhardt et al.	2015	USA	Not reported	Multidisciplinary outpatient clinicians	n/a	n/a	The CARE-D model recognizes the complexity of dementia syndromes and the unique needs of each person with dementia and the families. CARE-D builds a tailored care plan based on data from an individual’s psychosocial and neuropsychological assessments, relies on a skilled interdisciplinary team, and targets symptom-specific profiles, disease stage, and life stage.
Ollerenshaw	2015	Australia	Not reported	General physicians and nurses	n/a	n/a	The Dementia Pathways Tool provides information about region- specific, specialist dementia services and supports, together with current, accurate and relevant information about dementia to assist GPs in their practice, providing information to aid in assessment and diagnosis, referral, management and ongoing care. Access to an intuitive, online resource may also address the time restraints that some rural practitioners have identified as limiting their capacity to detect dementia.
Ollerenshaw et al.	2018	Australia	Cross sectional study	General physicians and nurses	42 (21 physicians, 21 nurses)	n/a	Online Dementia Pathway Tool provided rural and regional health practitioners access to clinical decision aids and region-specific referral and management resources for dementia. Findings suggest that the value of the pathway is closely connected to the content, the local resources and its perceived value to knowledge development and confidence. The Dementia Pathway Tool has the potential for wide ranging transferability to other health areas, particularly in rural and regional settings.
Petry et al.	2023	Switzerland	Mixed methods process evaluation	Nurses and clinical staff	72 (43 nurses, 29 clinical staff)	33.72 *±* 10.20	Organisational and process factors are the most influential determinants to the implementation and delivery of dementia care in acute care settings. The complexity of dementia care, along with the complexity of care environments, add to the difficulty of improving care delivery. This evaluation of a ‘failed implementation’ suggests that assessing and addressing organisational readiness in terms of available resources and implementation climate, such as compatibility of the ‘new’ intervention with existing care processes and culture, may provide the most leverage to improvement

*Abbreviations: N/A, not available

### Care pathway characteristics

#### Purpose

The aims of the care pathways are related to supporting those living with dementia, clinicians (i.e., primary care, specialists) or offering structured care processes within the clinical setting. For example, several studies articulated assisting persons with cognitive impairment with early diagnosis and treatment, including non-pharmacological and managed care [23, 41, 43, 46]. Another stated purpose concerned supporting clinicians in their timely and accurate decision-making processes to detect, diagnose, and treat dementia and to access information to support patients’ diagnosis, referral, and ongoing management [18, 21, 40, 42, 45]. Pathways embedded in dementia specialty care settings offered structure and clinical procedures for clinicians participating [47] and a collaborative care model [37, 44]. One study in their care pathway objective included providing service support for care partners of people with memory problems [41]. Another study indicated the pathway as a visual representation to inform the future redesign of dementia care delivery [39].

#### Design process

The design of the care pathways used different approaches. For several, co-design activities, reflections, or an inclusive management approach involving comprehensive consultation with stakeholders occurred to review and refine the proposed pathway [21, 23, 37–40, 43, 45]. Fitzgerald, Curry [39] used a “storyboard” design to depict how “consumers” and their carers would like to experience the dementia journey using a particular software, Essomenic™. The design of other pathways, such as the CARE-D model, relied on conceptual frameworks of psychosocial and rehabilitative interventions [47], input from a National Dementia Strategy [41], or consultant case reports [38] and peer reviews from dementia experts [37, 46]. Emerging evidence of Alzheimer’s disease as a clinical and biological construct informed the development of Hampel, Au [18] bio-marker clinical care pathway.

#### Expected users

The expected users for most dementia care pathways were clinicians. A combination of primary care or specialist physicians and nurses was identified in three studies [18, 21, 45], and dementia-trained nurses were solely mentioned in two studies (i.e., “Admiral Nurses” [23, 44]. The target users for the remaining seven pathways were multidisciplinary clinicians, clinical staff, and healthcare professionals [37, 38, 40, 41, 43, 46, 47]. Fitzgerald, Curry [39] reported a combination of policymakers, healthcare providers and research as potential users of the experiential care pathway. As a publicly accessible resource, the Dementia Pathways Tool is also available to the general public [45].

#### Core components

The core components of the dementia care pathways are categorized in the following way: *Assessment*: Cognitive assessments, medical history, physical examination, and care needs to assist in identifying persons with mild cognitive impairment and more advanced dementia [23, 37, 38, 41–43, 45, 46]. Hampel, Au [18] categorizes diagnostic work-up as first-line (primary care) and second-line (Alzheimer’s disease specialist) approaches guided by biomarkers and behavioural or functional changes in the person. *Dementia-specific treatment (pharmacological and non-pharmacological)*: Interventions consisted of medical and non-medical (i.e., person-centred communication and counselling, memory aids, home modifications, etc.) [18, 37, 40, 43, 44, 46, 47] with higher or lower tiered assistance, depending on the level of need [44]. In the dementia care pathway from Goeman, King [40], quick reference cards offer culturally appropriate guidance for engaging with culturally and linguistically diverse groups. *Referral*: Referral pathways, including websites, service directories, and other resources (i.e., home care services) [41, 42, 45] help provide additional support (in-house or community) [23, 45]. As for Hean, Nojeed [41], the “single point of access” care pathway enables individuals to link to integrated community services. *Family support and education*: Support and education for family members were also considered [37, 41, 43, 45, 47], including immediately following diagnosis disclosure [41]. For example, Morhardt, Weintraub [47] share communication tips and alternative responses to challenging behaviour for families. *Care coordination*: Six studies referred to care coordination and developing care plans [23, 37, 40, 42, 46, 47], allowing for consistent re-evaluation of the selected interventions [23, 46]. Community collaboration and community-based care were also embedded in two pathways, working closely with researchers and community-based organizations to utilize existing resources and improve outcomes [37, 44].

#### Outcome measures

In the included papers, only six reported on outcome data. The outcome measures used to evaluate the dementia care pathways consist of the *implementability* of the pathways. These measures assessed the acceptability, appropriateness, and feasibility of the care pathway [43, 46] and awareness and usage of the tool (i.e., number of identification and diagnoses of dementia and new registrations, referrals made, views of the tool, etc.) [21, 41, 44]. Indicators that target *patient and family*-reported outcomes include evaluating changes in the physical, emotional, and mental well-being of the person with dementia and their families [44], visit satisfaction questionnaires [37, 41], and family feedback [46]. Several studies included *clinician* outcome measures that reported on interprofessional effectiveness and satisfaction with the care pathway using the Team Fitness Test [37], communication practices between services [44], dementia knowledge and competence [21, 43, 46], and staff workload [46]. Last, none of the studies considered *system-level* outcome measures except for inappropriate hospital admissions [44].

#### Evaluative data

The available evaluative data in the included studies reported on the dementia care pathway scoring high acceptability and low feasibility with concerns about introducing into clinical routines [43]. Several studies captured improvements in knowledge, skills, and confidence about core dementia topics from clinician survey respondents and team member feedback, respectively [21, 37, 46]. Page views and time spent at the virtual care pathway site were reported for a digital tool [21]. Several studies collected positive feedback about the service guided by a pathway on accessibility, better coordination and continuity, quicker response rate to referrals, and good treatment for users [41]. Aguirre, Hilsabeck [37] demonstrated a reduction in the intervening time between initial appointment and diagnosis to 2 months compared to 14–15 months. Feedback was also received from family members who noted the pathways facilitated more in-depth engagement of the person with dementia within the family [46]. Higher numbers of people diagnosed with dementia at an earlier stage were an indicator of success for one study. One study reported a high workload for each team member solved by restructuring staff duties [46], and another reported a higher cost associated with the pathway [41].

### Methodological limitations

Several studies referenced methodological limitations in evaluating dementia care pathways. These limitations stemmed from an absence of a more robust evaluation (e.g., economic evaluation, subjective, randomized control trial) [18, 21, 37, 40, 43], a small sample size [21, 37, 43], and a lack of transferability to other forms of dementia [46] or practice settings [21, 42, 45].

### Implications for persons with dementia and their care partners

The noted benefits of the dementia care pathways described in the studies include facilitating close collaboration and engagement with affected persons and family members and providing them and staff with methods (i.e., non-pharmacological strategies) to manage and cope with the changes related to dementia [43, 46, 47]. Two care pathways benefit particular sub-groups: persons with frontal-temporal-lobe dementias (FTLDs) [38] and culturally and linguistically diverse people [40]. The authors supporting the development of a prison-based dementia care pathway also implied positive implications for the older prisoners receiving an equivalent service to what happens in the community [23]. The noted advantage to clinician pathway users is access to evidence-based guidelines to inform care processes such as screening and referral services [21, 42, 45].

### Involvement by persons with dementia and/or their care partners

The involvement of persons with dementia and their care partners in developing, implementing, and/or evaluating the care pathways varied based on what the authors reported. Participation in developing the care pathways occurred in a series of co-design activities or feedback delivered in a validation workshop with family members [23, 43]. Experiential data was collected through semi-structured interviews and Experience Group sessions with patient-carer dyads [37, 38] and care partners who provided and validated data [42]. Two papers rely on or call for direct input from end users in developing ideal care pathways in the future. First, Fitzgerald, Curry [39] “ideal state” journey modelled the pathway on the “consumer voice.” Next, in the paper by Hampel, Au [18], the authors describe the future state of a biomarker-informed clinical pathway and advocate for the collective involvement of persons affected and care partners’ perspectives in developing the “next generation” of clinical pathways. According to the authors, their engagement will provide essential insight into the existing gaps in health services.

## Discussion

This study aimed to map the existing evidence of dementia care pathways and to determine the extent of involvement by persons with dementia and/or their care partners in developing, implementing, and evaluating the care pathways. Our review, which included 13 unique care pathways, is the first to focus on synthesizing published care pathways in dementia, which adds to the knowledge base of other reviews targeting pathways *into* aging or dementia care services [48, 49]. Our findings suggest that pathways mainly assist dementia diagnostic and management practices for clinicians and offer structured care processes in clinical settings (i.e., referral and treatment pathways). For this reason, these pathways emphasized assessment and interventional post-diagnostic support, with less emphasis on community-based integrated dementia care. With rising service demand [50] and the preference of people with dementia to live at home as long as possible [51], robust community-based dementia care can provide and coordinate potential solutions.

Only one pathway mentioned voluntary or third-sector organizations as an option for providing ongoing activities and community engagement [23], and several had pathways inclusive of community-based care and resources (i.e., home care and Alzheimer’s Society) [41, 44]. While quality home care for community-dwelling people with dementia and their care partners is vital to their independence and quality of life [52], affected individuals require access to various community care services to meet their needs (e.g., informational and social support, advice, and peer support) [53]. As such, support from voluntary or third-sector organizations is also valued [19]. The growing interest in dementia-friendly communities [54, 55] also means that dementia care pathways require more widespread integration of formal and informal health and social care locally.

While few of the pathways were planned for use by persons with dementia and/or their care partners, many received input from individuals with lived experience during the design process. However, none of the pathways received feedback on their implementation or evaluation measures. This confirms a positive shift to involving people with lived experience in co-creating interventions [56], although the varying degrees of involvement suggest room for improvement. For example, evidence-based design principles or tools and recommendations for the dementia stage could support the involvement of persons with dementia in all aspects of the co-design process [57], including identifying patient-reported outcome measures (PROMs) to inform the success of dementia care pathways [58]. Our findings indicate that less than half of the papers included outcome data measuring pathway effectiveness, and fewer utilized PROMs. Often, clinicians favor symptoms or functional limitation measurements in contrast to patients valuing quality-of-life [59] and autonomy outcome measures [60]. To our knowledge, there is no established set of PROMs for dementia care pathways. Similarly, identification of the outcomes of the most importance to persons with dementia involved in a dementia care pathway is also limited [19]. Yet, involving older people in producing meaningful PROMs is possible [60, 61] and warrants exploration in persons at varying stages of dementia and their care partners. Notably, reliance on proxies or clinicians for measuring PROMs in the dementia patient population has been shown [58] due to errors in self-reported questionnaires associated with cognitive impairment [62]. Innovative approaches, like eye-tracking technology, are being explored [63] to address these challenges.

Another area needing more exploration and consideration is the cultural relevance of established and future dementia care pathways. Our findings revealed only one study that described a pathway embedded within an inclusive model of culturally sensitive support to assist people with dementia from culturally and linguistically diverse communities [40]. There are enormous gaps in the dementia evidence on racial and ethnic groups [64], including disparities in dementia diagnosis and care [65, 66]. Challenges navigating health systems pose additional difficulties for racialized dementia care partners [67]. For this reason, developing dementia care pathways that consider the cultural influences on service use and cultural perceptions of dementia are needed [68]. Other areas where the pathways did not exclusively target were end-of-life care and advanced stages of dementia. Given the variable disease progression of dementia, care pathways would benefit from a long-term palliative care approach for those with advanced stages of the disease. Models formed around one care delivery location [37, 41, 42, 47] could potentially contribute to fragmenting care when care needs change and require transitions in location or provider [69]. Examples of more integrated dementia care systems include the Care Ecosystem Collaborative Model [70], designed to augment existing healthcare services [71]. Models such as this could help people with dementia and caregiver dyads not only with advanced planning and behavioural support [72] but also with navigating newer pharmaceutical treatment options. Such medical advances paired with holistic care approaches will improve the care that those living with dementia receive during the dementia trajectory.

### Strengths and limitations

The strengths of this scoping review include an existing protocol, which was peer-reviewed by study authors before conducting the review. We also conducted an in-depth search strategy developed by an information specialist. Two independent reviewers double-screened at all stages of the screening process. Our review also has several limitations. Quality assessments were not conducted. Also, a broad definition of the dementia care pathway was used, and the grey literature was not reviewed. These limitations may have contributed to missing relevant studies. However, now that we have identified the empirical literature with sufficient research evidence, the next step for the field is to assess non-peer-reviewed data sources, including websites that feature local dementia care pathways.

## Conclusion

This scoping review demonstrated a growing evidence base on dementia care pathways to support the diagnosis and post-diagnostic dementia phases. Most existing pathways focused on supporting diagnostic assessments and dementia-specific management aspects with less attention to ongoing care coordination and community support. Future dementia care pathway development can seek the involvement of persons with dementia and care partners in designing, implementing and evaluating such pathways, ensuring that outcome measures may properly reflect the impact on persons with lived dementia experience and their care partners.

### Electronic supplementary material

Below is the link to the electronic supplementary material.

Supplementary Material 1

Supplementary Material 2

## Data Availability

The data supporting the conclusion of this article is included within the article.
