# Supplementary material for: Mapping the evidence on dementia care pathways – A scoping review
Source: BMC Geriatr. 2024 Aug 17;24:690. doi: 10.1186/s12877-024-05250-4 (PMC11330604; doi:10.1186/s12877-024-05250-4)
Supplement: Supplementary file 2 — Supplementary Material 2 [file 12877_2024_5250_MOESM2_ESM.docx]

# Search Methods Dementia Critical Pathways

Marianne Saragosa September 21, 2023

## Search Methods

A comprehensive search strategy was developed using a combination of database-specific subject headings and text words for the main concepts of dementia and care pathways. The dementia search string was from the Cochrane Collaboration.^[[1]](#footnote-1)^ Results were limited to humans and English language.

We searched the following databases on September 21, 2023: Ovid MEDLINE; Ovid Embase; Cochrane Database of Systematic Reviews (Ovid); Cochrane Central Register of Controlled Trials (Ovid); PsycINFO (Ovid); and CINAHL Ultimate(EBSCOhost). Please see Appendix I for all search strategies.

The reference lists of included publications were also searched and considered for inclusion.

## Appendix I

Ovid MEDLINE(R) ALL <1946 to September 20, 2023>

| **#** | **Searches** | **Results** | **Type** |  |  |  |
| --- | --- | --- | --- | --- | --- | --- |
|  | | | | | | |
| 1 | exp Dementia/ | 206959 | Advanced |  |  |  |
| 2 | Delirium/ | 12261 | Advanced |  |  |  |
| 3 | Wernicke Encephalopathy/ | 1904 | Advanced |  |  |  |
| 4 | Delirium, Dementia, Amnestic, Cognitive Disorders/ | 9773 | Advanced |  |  |  |
| 5 | dement*.mp. | 166791 | Advanced |  |  |  |
| 6 | alzheimer*.mp. | 204487 | Advanced |  |  |  |
| 7 | (lewy* adj2 bod*).mp. | 12748 | Advanced |  |  |  |
| 8 | deliri*.mp. | 24759 | Advanced |  |  |  |
| 9 | (chronic adj2 cerebrovascular).mp. | 815 | Advanced |  |  |  |
| 10 | ("organic brain disease" or "organic brain syndrome").mp. | 816 | Advanced |  |  |  |
| 11 | ("normal pressure hydrocephalus" and "shunt*").mp. | 1785 | Advanced |  |  |  |
| 12 | "benign senescent forgetfulness".mp. | 18 | Advanced |  |  |  |
| 13 | (cerebr* adj2 deteriorat*).mp. | 265 | Advanced |  |  |  |
| 14 | (cerebral* adj2 insufficient*).mp. | 94 | Advanced |  |  |  |
| 15 | (pick* adj2 disease).mp. | 4482 | Advanced |  |  |  |
| 16 | (creutzfeldt or jcd or cjd).mp. | 9371 | Advanced |  |  |  |
| 17 | huntington*.mp. | 22836 | Advanced |  |  |  |
| 18 | binswanger*.mp. | 609 | Advanced |  |  |  |
| 19 | korsako*.mp. | 1847 | Advanced |  |  |  |
| 20 | or/1-19 | 374744 | Advanced |  |  |  |
| 21 | Critical Pathways/ | 7853 | Advanced |  |  |  |
| 22 | (critical adj2 path?).mp. | 749 | Advanced |  |  |  |
| 23 | (critical adj2 pathway?).mp. | 14172 | Advanced |  |  |  |
| 24 | (care adj2 path?).mp. | 518 | Advanced |  |  |  |
| 25 | (care adj2 pathway?).mp. | 9032 | Advanced |  |  |  |
| 26 | carepathway?.mp. | 2 | Advanced |  |  |  |
| 27 | (care adj2 model?).mp. | 23832 | Advanced |  |  |  |
| 28 | (care adj2 trajector*).mp. | 761 | Advanced |  |  |  |
| 29 | (care adj2 framework?).mp. | 1506 | Advanced |  |  |  |
| 30 | (clinical adj2 path?).mp. | 748 | Advanced |  |  |  |
| 31 | (clinical adj2 pathway?).mp. | 7116 | Advanced |  |  |  |
| 32 | (care adj2 map*).mp. | 592 | Advanced |  |  |  |
| 33 | or/21-32 | 53330 | Advanced |  |  |  |
| 34 | 20 and 33 | 1444 | Advanced |  |  |  |
| 35 | animals/ not (animals/ and humans/) | 5122694 | Advanced |  |  |  |
| 36 | 34 not 35 | 1426 | Advanced |  |  |  |
| 37 | limit 36 to english language | 1341 | Advanced |  |  |  |
| 38 | remove duplicates from 37 | 1337 | Advanced |  |  |  |

Embase <1974 to 2023 September 20>

| **#** | **Searches** | **Results** | **Type** |  |  |  |
| --- | --- | --- | --- | --- | --- | --- |
|  | | | | | | |
| 1 | exp dementia/ | 444604 | Advanced |  |  |  |
| 2 | Lewy body/ | 8252 | Advanced |  |  |  |
| 3 | delirium/ | 35595 | Advanced |  |  |  |
| 4 | Wernicke encephalopathy/ | 3196 | Advanced |  |  |  |
| 5 | cognitive defect/ | 215484 | Advanced |  |  |  |
| 6 | dement*.mp. | 259952 | Advanced |  |  |  |
| 7 | alzheimer*.mp. | 305829 | Advanced |  |  |  |
| 8 | (lewy* adj2 bod*).mp. | 23143 | Advanced |  |  |  |
| 9 | deliri*.mp. | 47773 | Advanced |  |  |  |
| 10 | (chronic adj2 cerebrovascular).mp. | 1305 | Advanced |  |  |  |
| 11 | ("organic brain disease" or "organic brain syndrome").mp. | 3423 | Advanced |  |  |  |
| 12 | "supranuclear palsy".mp. | 10000 | Advanced |  |  |  |
| 13 | ("normal pressure hydrocephalus" and "shunt*").mp. | 2528 | Advanced |  |  |  |
| 14 | "benign senescent forgetfulness".mp. | 31 | Advanced |  |  |  |
| 15 | (cerebr* adj2 deteriorat*).mp. | 370 | Advanced |  |  |  |
| 16 | (cerebral* adj2 insufficient*).mp. | 120 | Advanced |  |  |  |
| 17 | (pick* adj2 disease).mp. | 7522 | Advanced |  |  |  |
| 18 | (creutzfeldt or jcd or cjd).mp. | 14247 | Advanced |  |  |  |
| 19 | huntington*.mp. | 38071 | Advanced |  |  |  |
| 20 | binswanger*.mp. | 956 | Advanced |  |  |  |
| 21 | korsako*.mp. | 2807 | Advanced |  |  |  |
| 22 | CADASIL.mp. | 3178 | Advanced |  |  |  |
| 23 | or/1-22 | 731795 | Advanced |  |  |  |
| 24 | clinical pathway/ | 9936 | Advanced |  |  |  |
| 25 | (critical adj2 path?).mp. | 1069 | Advanced |  |  |  |
| 26 | (critical adj2 pathway?).mp. | 9351 | Advanced |  |  |  |
| 27 | (care adj2 path?).mp. | 861 | Advanced |  |  |  |
| 28 | (care adj2 pathway?).mp. | 15325 | Advanced |  |  |  |
| 29 | carepathway?.mp. | 23 | Advanced |  |  |  |
| 30 | (care adj2 model?).mp. | 34341 | Advanced |  |  |  |
| 31 | (care adj2 trajector*).mp. | 1077 | Advanced |  |  |  |
| 32 | (care adj2 framework?).mp. | 1907 | Advanced |  |  |  |
| 33 | (clinical adj2 path?).mp. | 1063 | Advanced |  |  |  |
| 34 | (clinical adj2 pathway?).mp. | 18130 | Advanced |  |  |  |
| 35 | (care adj2 map*).mp. | 866 | Advanced |  |  |  |
| 36 | or/24-35 | 78255 | Advanced |  |  |  |
| 37 | 23 and 36 | 3068 | Advanced |  |  |  |
| 38 | (exp animals/ or exp animal experimentation/ or nonhuman/) not ((exp animals/ or exp animal experimentation/ or nonhuman/) and exp human/) | 7259743 | Advanced |  |  |  |
| 39 | 37 not 38 | 2991 | Advanced |  |  |  |
| 40 | limit 39 to english language | 2909 | Advanced |  |  |  |
| 41 | remove duplicates from 40 | 2872 | Advanced |  |  |  |

EBM Reviews - Cochrane Central Register of Controlled Trials

| **#** | **Searches** | **Results** | **Type** |  |  |  |
| --- | --- | --- | --- | --- | --- | --- |
|  | | | | | | |
| 1 | exp Dementia/ | 9084 | Advanced |  |  |  |
| 2 | Delirium/ | 1210 | Advanced |  |  |  |
| 3 | Wernicke Encephalopathy/ | 6 | Advanced |  |  |  |
| 4 | Delirium, Dementia, Amnestic, Cognitive Disorders/ | 241 | Advanced |  |  |  |
| 5 | dement*.mp. | 16768 | Advanced |  |  |  |
| 6 | alzheimer*.mp. | 13572 | Advanced |  |  |  |
| 7 | (lewy* adj2 bod*).mp. | 502 | Advanced |  |  |  |
| 8 | deliri*.mp. | 5369 | Advanced |  |  |  |
| 9 | (chronic adj2 cerebrovascular).mp. | 722 | Advanced |  |  |  |
| 10 | ("organic brain disease" or "organic brain syndrome").mp. | 153 | Advanced |  |  |  |
| 11 | ("normal pressure hydrocephalus" and "shunt*").mp. | 97 | Advanced |  |  |  |
| 12 | "benign senescent forgetfulness".mp. | 2 | Advanced |  |  |  |
| 13 | (cerebr* adj2 deteriorat*).mp. | 39 | Advanced |  |  |  |
| 14 | (cerebral* adj2 insufficient*).mp. | 2 | Advanced |  |  |  |
| 15 | (pick* adj2 disease).mp. | 127 | Advanced |  |  |  |
| 16 | (creutzfeldt or jcd or cjd).mp. | 66 | Advanced |  |  |  |
| 17 | huntington*.mp. | 794 | Advanced |  |  |  |
| 18 | binswanger*.mp. | 7 | Advanced |  |  |  |
| 19 | korsako*.mp. | 76 | Advanced |  |  |  |
| 20 | or/1-19 | 31160 | Advanced |  |  |  |
| 21 | Critical Pathways/ | 265 | Advanced |  |  |  |
| 22 | (critical adj2 path?).mp. | 53 | Advanced |  |  |  |
| 23 | (critical adj2 pathway?).mp. | 362 | Advanced |  |  |  |
| 24 | (care adj2 path?).mp. | 44 | Advanced |  |  |  |
| 25 | (care adj2 pathway?).mp. | 1050 | Advanced |  |  |  |
| 26 | carepathway?.mp. | 0 | Advanced |  |  |  |
| 27 | (care adj2 model?).mp. | 3894 | Advanced |  |  |  |
| 28 | (care adj2 trajector*).mp. | 58 | Advanced |  |  |  |
| 29 | (care adj2 framework?).mp. | 94 | Advanced |  |  |  |
| 30 | (clinical adj2 path?).mp. | 82 | Advanced |  |  |  |
| 31 | (clinical adj2 pathway?).mp. | 842 | Advanced |  |  |  |
| 32 | (care adj2 map*).mp. | 94 | Advanced |  |  |  |
| 33 | or/21-32 | 6126 | Advanced |  |  |  |
| 34 | 20 and 33 | 250 | Advanced |  |  |  |
| 35 | limit 34 to english language | 249 | Advanced |  |  |  |
| 36 | remove duplicates from 35 | 245 | Advanced |  |  |  |
| EBM Reviews - Cochrane Database of Systematic Reviews <2005 to September 20, 2023>   \| **#** \| **Searches** \| **Results** \| **Type** \| \| --- \| --- \| --- \| --- \| \|  \| \| \| \| \| \| \| \| 1 \| dement*.ti,ab. \| 240 \| Advanced \|  \|  \|  \| \| 2 \| alzheimer*.ti,ab. \| 101 \| Advanced \|  \|  \|  \| \| 3 \| (lewy* adj2 bod*).ti,ab. \| 11 \| Advanced \|  \|  \|  \| \| 4 \| deliri*.ti,ab. \| 41 \| Advanced \|  \|  \|  \| \| 5 \| (chronic adj2 cerebrovascular).ti,ab. \| 2 \| Advanced \|  \|  \|  \| \| 6 \| ("organic brain disease" or "organic brain syndrome").ti,ab. \| 0 \| Advanced \|  \|  \|  \| \| 7 \| "supranuclear palsy".ti,ab. \| 2 \| Advanced \|  \|  \|  \| \| 8 \| ("normal pressure hydrocephalus" and "shunt*").ti,ab. \| 4 \| Advanced \|  \|  \|  \| \| 9 \| "benign senescent forgetfulness".ti,ab. \| 0 \| Advanced \|  \|  \|  \| \| 10 \| (cerebr* adj2 deteriorat*).ti,ab. \| 0 \| Advanced \|  \|  \|  \| \| 11 \| (cerebral* adj2 insufficient*).ti,ab. \| 1 \| Advanced \|  \|  \|  \| \| 12 \| (pick* adj2 disease).ti,ab. \| 0 \| Advanced \|  \|  \|  \| \| 13 \| (creutzfeldt or jcd or cjd).ti,ab. \| 2 \| Advanced \|  \|  \|  \| \| 14 \| huntington*.ti,ab. \| 4 \| Advanced \|  \|  \|  \| \| 15 \| binswanger*.ti,ab. \| 0 \| Advanced \|  \|  \|  \| \| 16 \| korsako*.ti,ab. \| 2 \| Advanced \|  \|  \|  \| \| 17 \| or/1-16 \| 285 \| Advanced \|  \|  \|  \| \| 18 \| (critical adj2 path?).ti,ab. \| 0 \| Advanced \|  \|  \|  \| \| 19 \| (critical adj2 pathway?).ti,ab. \| 0 \| Advanced \|  \|  \|  \| \| 20 \| (care adj2 path?).ti,ab. \| 0 \| Advanced \|  \|  \|  \| \| 21 \| (care adj2 pathway?).ti,ab. \| 7 \| Advanced \|  \|  \|  \| \| 22 \| carepathway?.ti,ab. \| 0 \| Advanced \|  \|  \|  \| \| 23 \| (care adj2 model?).ti,ab. \| 38 \| Advanced \|  \|  \|  \| \| 24 \| (care adj2 trajector*).ti,ab. \| 0 \| Advanced \|  \|  \|  \| \| 25 \| (care adj2 framework?).ti,ab. \| 1 \| Advanced \|  \|  \|  \| \| 26 \| (clinical adj2 path?).ti,ab. \| 0 \| Advanced \|  \|  \|  \| \| 27 \| (clinical adj2 pathway?).ti,ab. \| 21 \| Advanced \|  \|  \|  \| \| 28 \| (care adj2 map*).ti,ab. \| 0 \| Advanced \|  \|  \|  \| \| 29 \| or/18-28 \| 66 \| Advanced \|  \|  \|  \| \| 30 \| 17 and 29 \| 6 \| Advanced \|  \|  \|  \|   APA PsycInfo <1806 to September Week 2 2023>   \| **#** \| **Searches** \| **Results** \| **Type** \| \| --- \| --- \| --- \| --- \| \|  \| \| \| \| \| \| \| \| 1 \| exp Dementia/ \| 92010 \| Advanced \|  \|  \|  \| \| 2 \| exp Delirium/ \| 4131 \| Advanced \|  \|  \|  \| \| 3 \| exp Huntingtons Disease/ \| 3571 \| Advanced \|  \|  \|  \| \| 4 \| exp Kluver Bucy Syndrome/ \| 58 \| Advanced \|  \|  \|  \| \| 5 \| exp Wernickes Syndrome/ \| 291 \| Advanced \|  \|  \|  \| \| 6 \| exp Cognitive Impairment/ \| 44588 \| Advanced \|  \|  \|  \| \| 7 \| dement*.mp. \| 91059 \| Advanced \|  \|  \|  \| \| 8 \| alzheimer*.mp. \| 77276 \| Advanced \|  \|  \|  \| \| 9 \| (lewy* adj2 bod*).mp. \| 4963 \| Advanced \|  \|  \|  \| \| 10 \| deliri*.mp. \| 9298 \| Advanced \|  \|  \|  \| \| 11 \| (chronic adj2 cerebrovascular).mp. \| 107 \| Advanced \|  \|  \|  \| \| 12 \| ("organic brain disease" or "organic brain syndrome").mp. \| 879 \| Advanced \|  \|  \|  \| \| 13 \| "supranuclear palsy".mp. \| 1720 \| Advanced \|  \|  \|  \| \| 14 \| ("normal pressure hydrocephalus" and "shunt*").mp. \| 261 \| Advanced \|  \|  \|  \| \| 15 \| "benign senescent forgetfulness".mp. \| 28 \| Advanced \|  \|  \|  \| \| 16 \| (cerebr* adj2 deteriorat*).mp. \| 52 \| Advanced \|  \|  \|  \| \| 17 \| (cerebral* adj2 insufficient*).mp. \| 6 \| Advanced \|  \|  \|  \| \| 18 \| (pick* adj2 disease).mp. \| 842 \| Advanced \|  \|  \|  \| \| 19 \| (creutzfeldt or jcd or cjd).mp. \| 1253 \| Advanced \|  \|  \|  \| \| 20 \| huntington*.mp. \| 5603 \| Advanced \|  \|  \|  \| \| 21 \| binswanger*.mp. \| 521 \| Advanced \|  \|  \|  \| \| 22 \| korsako*.mp. \| 1372 \| Advanced \|  \|  \|  \| \| 23 \| or/1-22 \| 169368 \| Advanced \|  \|  \|  \| \| 24 \| (critical adj2 path?).mp. \| 135 \| Advanced \|  \|  \|  \| \| 25 \| (critical adj2 pathway?).mp. \| 779 \| Advanced \|  \|  \|  \| \| 26 \| (care adj2 path?).mp. \| 93 \| Advanced \|  \|  \|  \| \| 27 \| (care adj2 pathway?).mp. \| 1825 \| Advanced \|  \|  \|  \| \| 28 \| carepathway?.mp. \| 0 \| Advanced \|  \|  \|  \| \| 29 \| (care adj2 model?).mp. \| 7867 \| Advanced \|  \|  \|  \| \| 30 \| (care adj2 trajector*).mp. \| 286 \| Advanced \|  \|  \|  \| \| 31 \| (care adj2 framework?).mp. \| 623 \| Advanced \|  \|  \|  \| \| 32 \| (clinical adj2 path?).mp. \| 87 \| Advanced \|  \|  \|  \| \| 33 \| (clinical adj2 pathway?).mp. \| 589 \| Advanced \|  \|  \|  \| \| 34 \| (care adj2 map*).mp. \| 225 \| Advanced \|  \|  \|  \| \| 35 \| or/24-34 \| 11985 \| Advanced \|  \|  \|  \| \| 36 \| 23 and 35 \| 844 \| Advanced \|  \|  \|  \| \| 37 \| limit 36 to english language \| 792 \| Advanced \|  \|  \|  \| \| 38 \| remove duplicates from 37 \| 792 \| Advanced \|  \|  \|  \| | | |  |  | | |

| **#** | **Query** | **Limiters/Expanders** | **Last Run Via** | **Results** |
| --- | --- | --- | --- | --- |
| S1 | (MH "Dementia+") | Expanders - Apply equivalent subjects  Search modes - Boolean/Phrase | Interface - EBSCOhost Research Databases  Search Screen - Advanced Search  Database - CINAHL Ultimate | 85,557 |
| S2 | (MH "Delirium") or (MH "Delirium, Dementia, Amnestic, Cognitive Disorders") | Expanders - Apply equivalent subjects  Search modes - Boolean/Phrase | Interface - EBSCOhost Research Databases  Search Screen - Advanced Search  Database - CINAHL Ultimate | 8,513 |
| S3 | (MH "Wernicke's Encephalopathy") | Expanders - Apply equivalent subjects  Search modes - Boolean/Phrase | Interface - EBSCOhost Research Databases  Search Screen - Advanced Search  Database - CINAHL Ultimate | 486 |
| S4 | TX dement* | Expanders - Apply equivalent subjects  Search modes - Boolean/Phrase | Interface - EBSCOhost Research Databases  Search Screen - Advanced Search  Database - CINAHL Ultimate | 185,266 |
| S5 | TX alzheimer* | Expanders - Apply equivalent subjects  Search modes - Boolean/Phrase | Interface - EBSCOhost Research Databases  Search Screen - Advanced Search  Database - CINAHL Ultimate | 126,452 |
| S6 | TX lewy* N2 bod* | Expanders - Apply equivalent subjects  Search modes - Boolean/Phrase | Interface - EBSCOhost Research Databases  Search Screen - Advanced Search  Database - CINAHL Ultimate | 7,853 |
| S7 | TX deliri* | Expanders - Apply equivalent subjects  Search modes - Boolean/Phrase | Interface - EBSCOhost Research Databases  Search Screen - Advanced Search  Database - CINAHL Ultimate | 31,646 |
| S8 | TX chronic N2 cerebrovascular | Expanders - Apply equivalent subjects  Search modes - Boolean/Phrase | Interface - EBSCOhost Research Databases  Search Screen - Advanced Search  Database - CINAHL Ultimate | 1,215 |
| S9 | TX "organic brain disease" or "organic brain syndrome" | Expanders - Apply equivalent subjects  Search modes - Boolean/Phrase | Interface - EBSCOhost Research Databases  Search Screen - Advanced Search  Database - CINAHL Ultimate | 530 |
| S10 | TX "normal pressure hydrocephalus" and "shunt*" | Expanders - Apply equivalent subjects  Search modes - Boolean/Phrase | Interface - EBSCOhost Research Databases  Search Screen - Advanced Search  Database - CINAHL Ultimate | 286 |
| S11 | TX "benign senescent forgetfulness" | Expanders - Apply equivalent subjects  Search modes - Boolean/Phrase | Interface - EBSCOhost Research Databases  Search Screen - Advanced Search  Database - CINAHL Ultimate | 46 |
| S12 | TX cerebr* N2 deteriorat* | Expanders - Apply equivalent subjects  Search modes - Boolean/Phrase | Interface - EBSCOhost Research Databases  Search Screen - Advanced Search  Database - CINAHL Ultimate | 244 |
| S13 | TX cerebral* N2 insufficient* | Expanders - Apply equivalent subjects  Search modes - Boolean/Phrase | Interface - EBSCOhost Research Databases  Search Screen - Advanced Search  Database - CINAHL Ultimate | 88 |
| S14 | TX pick* N2 disease | Expanders - Apply equivalent subjects  Search modes - Boolean/Phrase | Interface - EBSCOhost Research Databases  Search Screen - Advanced Search  Database - CINAHL Ultimate | 1,801 |
| S15 | TX creutzfeldt or jcd or cjd | Expanders - Apply equivalent subjects  Search modes - Boolean/Phrase | Interface - EBSCOhost Research Databases  Search Screen - Advanced Search  Database - CINAHL Ultimate | 3,736 |
| S16 | TX huntington* | Expanders - Apply equivalent subjects  Search modes - Boolean/Phrase | Interface - EBSCOhost Research Databases  Search Screen - Advanced Search  Database - CINAHL Ultimate | 36,109 |
| S17 | TX binswanger* | Expanders - Apply equivalent subjects  Search modes - Boolean/Phrase | Interface - EBSCOhost Research Databases  Search Screen - Advanced Search  Database - CINAHL Ultimate | 1,118 |
| S18 | TX korsako* | Expanders - Apply equivalent subjects  Search modes - Boolean/Phrase | Interface - EBSCOhost Research Databases  Search Screen - Advanced Search  Database - CINAHL Ultimate | 1,567 |
| S19 | S1 OR S2 OR S3 OR S4 OR S5 OR S6 OR S7 OR S8 OR S9 OR S10 OR S11 OR S12 OR S13 OR S14 OR S15 OR S16 OR S17 OR S18 | Expanders - Apply equivalent subjects  Search modes - Boolean/Phrase | Interface - EBSCOhost Research Databases  Search Screen - Advanced Search  Database - CINAHL Ultimate | 298,489 |
| S20 | (MH "Critical Path") | Expanders - Apply equivalent subjects  Search modes - Boolean/Phrase | Interface - EBSCOhost Research Databases  Search Screen - Advanced Search  Database - CINAHL Ultimate | 6,047 |
| S21 | TI (critical N2 path#) OR AB (critical N2 path#) | Expanders - Apply equivalent subjects  Search modes - Boolean/Phrase | Interface - EBSCOhost Research Databases  Search Screen - Advanced Search  Database - CINAHL Ultimate | 449 |
| S22 | TI (critical N2 pathway#) OR AB (critical N2 pathway#) | Expanders - Apply equivalent subjects  Search modes - Boolean/Phrase | Interface - EBSCOhost Research Databases  Search Screen - Advanced Search  Database - CINAHL Ultimate | 1,269 |
| S23 | TI (care N2 path#) OR AB (care N2 path#) | Expanders - Apply equivalent subjects  Search modes - Boolean/Phrase | Interface - EBSCOhost Research Databases  Search Screen - Advanced Search  Database - CINAHL Ultimate | 472 |
| S24 | TI (care N2 pathway#) OR AB (care N2 pathway#) | Expanders - Apply equivalent subjects  Search modes - Boolean/Phrase | Interface - EBSCOhost Research Databases  Search Screen - Advanced Search  Database - CINAHL Ultimate | 6,085 |
| S25 | TI carepathway# OR AB carepathway# | Expanders - Apply equivalent subjects  Search modes - Boolean/Phrase | Interface - EBSCOhost Research Databases  Search Screen - Advanced Search  Database - CINAHL Ultimate | 4 |
| S26 | TI (care N2 model#) OR AB (care N2 model#) | Expanders - Apply equivalent subjects  Search modes - Boolean/Phrase | Interface - EBSCOhost Research Databases  Search Screen - Advanced Search  Database - CINAHL Ultimate | 22,846 |
| S27 | TI (care N2 trajector*) OR AB (care N2 trajector*) | Expanders - Apply equivalent subjects  Search modes - Boolean/Phrase | Interface - EBSCOhost Research Databases  Search Screen - Advanced Search  Database - CINAHL Ultimate | 747 |
| S28 | TI (care N2 framework#) OR AB (care N2 framework#) | Expanders - Apply equivalent subjects  Search modes - Boolean/Phrase | Interface - EBSCOhost Research Databases  Search Screen - Advanced Search  Database - CINAHL Ultimate | 2,348 |
| S29 | TI (clinical N2 path#) OR AB (clinical N2 path#) | Expanders - Apply equivalent subjects  Search modes - Boolean/Phrase | Interface - EBSCOhost Research Databases  Search Screen - Advanced Search  Database - CINAHL Ultimate | 341 |
| S30 | TI (clinical N2 pathway#) OR AB (clinical N2 pathway#) | Expanders - Apply equivalent subjects  Search modes - Boolean/Phrase | Interface - EBSCOhost Research Databases  Search Screen - Advanced Search  Database - CINAHL Ultimate | 3,592 |
| S31 | TI (care N2 map*) OR AB (care N2 map*) | Expanders - Apply equivalent subjects  Search modes - Boolean/Phrase | Interface - EBSCOhost Research Databases  Search Screen - Advanced Search  Database - CINAHL Ultimate | 705 |
| S32 | S20 OR S21 OR S22 OR S23 OR S24 OR S25 OR S26 OR S27 OR S28 OR S29 OR S30 OR S31 | Expanders - Apply equivalent subjects  Search modes - Boolean/Phrase | Interface - EBSCOhost Research Databases  Search Screen - Advanced Search  Database - CINAHL Ultimate | 39,657 |
| S33 | S19 AND S32 | Expanders - Apply equivalent subjects  Search modes - Boolean/Phrase | Interface - EBSCOhost Research Databases  Search Screen - Advanced Search  Database - CINAHL Ultimate | 2,653 |
| S34 | S19 AND S32 | Expanders - Apply equivalent subjects  Narrow by Language: - english  Search modes - Boolean/Phrase | Interface - EBSCOhost Research Databases  Search Screen - Advanced Search  Database - CINAHL Ultimate | 2,599 |

1. Woods, Bob, Rai, Kaur Harleen, Elliott, Emma, Aguirre, Elisa, Orrell, Martin, Spector, Aimee. Cognitive stimulation to improve cognitive functioning in people with dementia. Cochrane Database of Systematic Reviews. 2023;(1). Available from Cochrane Database of Systematic Reviews at http://ovidsp.ovid.com/ovidweb.cgi?T=JS&PAGE=reference&D=coch&NEWS=N&AN=00075320-100000000-04539. Accessed September 21, 2023. [↑](#footnote-ref-1)
